# Selections and Abstracts

**Published:** 1902-12

**Authors:** 


### SELECTIONS AND ABSTRACTS.

### The Management of Normal Labor, with Additional Remarks Regarding the Obstetrical Forceps.*

In obstetric practice, as well as in every other branch of medicine, physicians generally hold different opinions as to the best mode of conducting their cases. This is due no doubt to the teaching received in different universities and also to the fact that as each one acquires experience, he treats his cases according to his best judgment.

There is no class of cases with which the physician has to deal that calls for more sincere and conservative work than do those met with in obstetric practice, as in many instances the lives of both mother and child are partly, if not wholly, dependent on the judgment used by the physician in charge; therefore it becomes his duty to terminate labor in such a way as to prevent injury to either, or in cases where this is found impossible, to reduce the injuries to a minimum. To do this, he must know when and how to act, as well as when to abstain, the latter being very essential, as many of the injuries caused are due to too early interference.

There is probably no other question harder to decide in obstetric practice than to know when the physiological limits have been reached and that further waiting is more dangerous than interference. Conditions may arise in the apparently simple case that call for active and quick interference; therefore the physician should always go prepared to meet and combat any emergency that may arise. The woman's temperament should be studied in each case, as well as her power of resisting pain. Any physician who has attended a hysterical primipara knows well the firmness with which he must act in order to abstain from too early interference. The woman may be told that her first labor should not be hurried and that it should extend over a period of at least

*Read before the Nebraska State Medical Society, Omaha, Neb., May 6-8, 1902, by J. J. Cameron, M.D., Kearney, Neb.

twenty hours. This procedure sometimes saves the physician a great deal of annoyance from friends and neighboring women usually present at such cases, and who are many times directly responsible for too early interference on the part of the physician. Labor pains are judged from the effects they produce and not from the amount of suffering entailed on the patient, and should in a normal labor be sufficiently strong to overcome the resistance met with. They should be of an expulsive character and should have sufficient rest between each pain to allow the uterine muscle to rest and the nervous system to recover from the shock of the pain produced.

At the first visit the patient and her surroundings are made as clean as conditions will allow. After the hands have been made clean the first examination is made to determine the presentation we are about to deal with and to inform those interested as to the patient's condition and the probable duration of labor. Regarding the latter the physician's statement should be guarded. During the first stage very few examinations should be made, probably not more than two or three where normal conditions exist, for the woman's safety from puerperal sepsis depends largely on the fewness of digital examinations made. In many cases, especially in the country, we find that several examinations have been made by an incompetent nurse before the arrival of the physician. This complicates the conditions the physician has to deal with and should be stopped by telling the woman, where we have had a chance to make a previous visit, not to allow any one to make an examination before the arrival of the physician. In country practice the majority of cases we know nothing of until called at the beginning of labor, and although our patients should be taught that it is best for the woman's safety to have the physician called a month or two previous to confinement, still the physician cannot well refuse when called upon in emergency.

The first stage should be made as easy as possible for the patient, and in primiparæ lasts about ten or twelve hours, during which time the pains are sharp and irritating. Three or four one-grain opium powders may be left, one to be given every one or two hours as required. The patient will usually pass through this stage with very little pain, better able to withstand the nervous strain that usually accompanies it, and saves her strength until the expulsion stage has been reached. The patient may be warned against bearing down in this stage, as using her strength without gaining any material advantage. It is not advisable for the physician to remain at the house through this stage, as it might result in the too early use of the forceps against his better judgment. This is especially true in cases of primiparæ.

In multiparæ the first stage is usually well advanced or complete at the first visit.

Vertex presentations alone are considered normal.

The left occipital anterior occurs in about 70 per cent. of cases.

The right occipital posterior occurs in about 27 per cent. of cases.

The left occipital posterior occurs in about 1 per cent. of cases.

The right occipital anterior occurs in about 2 per cent. of cases.

In either one of these four positions the occiput usually rotates to the front and appears under the pubes, except in cases where the child's head is small and the pelvis roomy.

When the first stage is completed the amniotic sac has performed its function in labor and usually ruptures. In cases where this does not occur the membrane should be punctured before it has descended half the length of the vagina, as after this it retards rather than assists labor.

Throughout the second stage the physician should be constantly in attendance, the patient may be told to empty the bladder and rectum as often as possible, and in case of retention the catheter should be used and a rectal enema given to clear out the lower bowel. Attending to the bladder before and after confinement is very essential. A full bladder will lessen the strength of the pains in the second stage and delay labor; besides this there is danger of rupture from overdistention. As I know of this accident happening within the last month with a fatal termination, I concluded to at least refer to it. (I may say that it did not occur within the practice of a physician eligible to membership in this society.) More examinations may be made during the second stage, and some of these should be made during a pain to ascertain the strength of the pains and the progress being made. In cases where labor is slow at this time, the woman being tired, which is usually associated with weak uterine pains, it is not advisable to give anything to excite uterine contractions, neither should the forceps be used before first giving the patient a cup of tea, coffee, or broth and following this with bromide of potassium 20 grains, chloral hydrate 10 grains, where the heart will permit, or a hypodermic of morphia and allow to sleep for an hour. On awakening she will usually be found in a much better condition, the pains are renewed with increased vigor and frequency, and labor terminated in a large majority of cases in a natural way. I have never practiced pressure to the breech through the fundus in delayed labor, as a full uterus, in my opinion, might be easily ruptured in this way, and where the above procedure fails I prefer the careful use of the forceps to expression.

The chief duty of the physician in the second stage of labor is to prevent perineal rupture. The safety of the perineum depends largely on its elasticity. This may be increased by applying hot fomentations of a bichloride solution, 1 to 1000 strength, every minute for fifteen minutes before expulsion of the head. The perineum is richly supplied with blood-vessels, the hot applications prevent venous stasis from pressure, change the color, and make the perineum much more distensible.

The head is made to pass the vulva by its smallest diameter. This is accomplished by causing increased flexion with the fore-fingers pressing upward and backward on the anterior part of the head, while the thumb is used in directing the occiput forward under the pubic arch. The head is forced downward during each uterine contraction and again recedes during the interval following. This procedure should be carried on as long as possible, as it assists in softening the perineum. The head should not be allowed to pass the vulva when it would (if not interfered with). It should be held back for two or three pains, when it will be found that flexion is increased and the occiput advanced further under the pubic arch, while the head can be released at the will of the operator between pains. Undoubtedly perineal ruptures are lessened by holding back the head instead of supporting the perineum. The head should be closely watched during the last two or three pains, as perineal rupture is more likely to occur at this time. An anesthetic has probably its most value in labor when the head commences to distend the perineum and vulva. I have made a practice of giving it in every case at this time where the heart will permit. The chloroform of A. C. E. mixture may be given freely and the patient put quite asleep, a condition which she reaches easily, if told to breathe quickly with her mouth open. This keeps her from holding her breath and bearing down and lessens the danger of a too rapid expulsion of the head. The chloroform is given well diluted with air. When this precaution is taken it is rare that any bad results follow.

The anterior arm and shoulder is next delivered and should be raised well up against the pubic arch to lessen the danger of rupture by the posterior shoulder. From the time the shoulders are delivered there should be constant friction made on the fundus until the placenta is expelled to guard against relaxation and hemorrhage. The uterus is a bipolar organ, and by keeping the fundus contracted the cervix remains open and the placenta is more readily removed.

I have made a practice of not tying the cord until the pulsations have nearly ceased, except in cases of emergency. The changing of the child's circulation cannot be expected to take place in a moment's time. Early tying of the cord is apt to deprive the child of blood required to fill the pulmonary vessels.

Severe pressure should not be made on the uterus to expel the placenta without first tying the cord, as cardiac rupture might take place by forcing too much blood into the child's circulation.

There is usually a resting period of from five to ten minutes after the birth of the child before the uterus again begins to contract, during which time a tonic contraction should be maintained. When the uterine contraction returns, the placenta may be expelled about the third or fourth pain by the Credé method, together with slight traction on the cord. When a tonic contraction has been maintained in the uterus after the birth of the child, and where traction has not been made on the cord to remove the placenta during uterine relaxation, the condition known as "hour-glass contraction" will rarely occur. Under no consideration should there be pressure made on the fundus or traction made on the cord during the absence of uterine contraction, as inversion might occur.

Ergot may be given after expulsion of the placenta, and, if post-partum hemorrhage is threatened, an injection of ergotine deep into the gluteal muscles. If hemorrhage occurs before the placenta has been expelled, the uterus should be rapidly emptied. Probably the first indication we have of an approaching post-partum hemorrhage is a rapid rise in the heart-beat, the pulse running from 100 to 120, and should be a warning to the physician to act quickly.

It is well for the physician to remain at the house for at least an hour after the completion of labor, and before leaving should give the woman a hot antiseptic douche; it is more cleanly and serves in removing soreness.

The bandage, to be of much use and comfort, should extend from below the trochanters to the upper part of the abdomen, completely enveloping the pelvis, the ligaments and cartilages of which must have been necessarily stretched during the passage of the child. A comfortably tight bandage will surely assist in restoring both the bones of the pelvis and tissues of the abdomen to their original state and should be applied after labor has been completed and worn for at least ten or twelve days.

### FORCEPS.

The obstetrical forceps are for use in cases where in the judgment of the physician nature is about to fail in completing labor, or in cases where complications exist which necessitate quick delivery in the interest of either mother or child. Unluckily it has not always been confined to cases of this nature. I have known the instruments to be used within three hours of the beginning of a normal labor, and no doubt many others present have seen similar cases. The forceps have saved the lives of many children that otherwise would have perished from compression in a narrow pelvis, but it has also been used many times unnecessarily to the detriment of many others that would have been born in a natural way if only given the proper time to do so. It has also been of unmeasurable assistance to many mothers, but has made equally as many invalids for life by its too early and forceful use. The object of the physician when called upon to use the forceps should be to obtain the maximum of good with the minimum of harm. A great deal depends upon the skill with which it is used. As a physician acquires experience he lessens the chances of injury to both mother and child.

I have made a practice where no complications exist, to abstain from the use of the forceps until after the woman has been in labor from seventeen to twenty hours where the labor is a first one, and from ten to twelve hours for the second or subsequent labors.

The forceps should not be used as a means of saving the physician's time. Force should not be used in applying the forceps; it is not necessary if the blades are allowed to follow the pelvic curve. After the blades have been introduced, traction should not be made before first examining the cervix, to be sure it is not contained within the blades. There are cases where this accident might happen, especially in primiparæ. The membranes having ruptured early, the anterior wall of the cervix may become so thin from pressure that on examination one is led to believe that dilatation is complete and that he is feeling the uncovered head, but on examining further up and back toward the sacrum the undilated os may be found. Anteversion is usually present in those cases. I had such a case not long since in a primipara and was completely misled as to the time labor would be completed. Instead of ending in three hours, as I had expected, the labor was completed in a natural way twelve hours later. I mention this case because it was one that might have led to the too early use of the forceps and with bad results.

The membranes should be freely ruptured before applying the instruments, so as to prevent premature detachment of the placenta.

When it becomes necessary to use the forceps, the woman is anesthetized and may be placed on her left side, or better still, in the dorsal position, and the hips brought to the edge of the bed, with the limbs held by two assistants. Traction should be made at first with very little force, and made to simulate the natural pains as much as possible, as well as in conjunction with the latter, where it is possible, and should last about one minute, with a rest of one to one and a half minutes before again applying traction.

As the forceps, no matter how delicately the blades may be constructed, increase the diameter of the head, the blades should be removed before the largest diameter of the head reaches the vulva, but not before the chin of the child can be reached by the left forefinger in the rectum, to prevent the head receding. At this time the head will be found under almost full control of the physician and may be released at will. The forceps, by elevating the handles, will lift the head off the perineum and lessen the danger of rupture, as well as preventing a too rapid expulsion of the head.

The injuries usually caused by the too early and forceful use of the forceps are generally limited to the cervix, vagina, perineum and head of the child. Besides these there is the danger of applying traction during the absence of uterine contraction, as inversion might occur. In cases where the cord is abnormally short or is around the child's neck several times, this accident is sometimes unavoidable.

In conclusion, I may say that there are many other things that might have been said on the subject of this paper, but what has been said may be of some benefit to those who may be starting their medical career, while those who are older in experience may approve or condemn what has been said, in the light of their greater knowledge of the subject.— *Western Medical Review.*

---

### OBSERVATIONS ON ANESTHESIA OF THE DRUM MEMBRANE.*

The majority of clinicians do not believe in trying to obtain local anesthesia of the membrana tympani. Their deductions have been drawn in the main from the futility of using cocain for this purpose in the external auditory meatus. It is but rational to believe that Nature protects the tympanic cavity from the effects of fluids dropped by chance or design into the external canal. This protection is given by the dermal layer of the drum membrane—a skin without glandular action or hair, acting only as a shield for the layers beneath.

*Read before American Otological Society at New London, Conn., July 7, 1902, by Geo. B McAuliffe, A.B., M.D. Oculist and Aurist, Red Cross Hospital, Northwestern Dispensary, Harlem Hospital, N. Y. Mothers' Home; Con. Aurist Metropolitan Throat Hospital. Adj. Professor Otology, N. Y. Polyclinic; Assistant Aural Surgeon, Manhattan Eye and Ear Hospital.

Jacques, by utilizing the selective action of methylene blue, mapped out the nerve plexus in the middle layer of the drum membrane. The nerves spread out in radial meshes from the periphery—mostly from above. In the deeper portion of the dermal layer detached bundles run in different directions and end in apparently sensory end tips.

The mucous membrane of the Eustachian tube and of the tympanic cavity get their main nervous supply from the same source—the glossopharyngeal.

From a consideration of these facts we see that the external dermal layer has very little to do with the sensitivity of the drum membrane, and that most of the medicines dropped into the ear or applied to the drum membrane have little effect until they nullify the shieldlike action of the skin covering.

The fact that refrigeration does not extend deeply enough to desensitize the membrane demonstrates the truth of the former of the above mentioned conclusions. Furthermore it cannot be localized to the track of the intended incision. The refrigerating sprays need a space of a few inches to secure evaporation. This would bring under its action the whole membrane and canal. I tried to get a tip devised for spraying ethyl chloride on the region of the membrane selected for operation but was not successful. The application of the spray to the sensitive canal and the subsequent thawing are very painful. I have thought that if liquid air could be applied, as it is claimed, by a cotton applicator, it would be the ideal refrigerant knife for the membrana tympani. Unfortunately, too, refrigerants interfere with healing and may cause sloughing.

Various preparations like Bonain's—menthol, carbolic acid and cocain—depending for their action principally on the carbolic acid, have been used. More or less success has been reported. I do not believe that the anesthesia obtained by this class of cauterants is ever complete for reasons given above.

Fluids which disturb the osmotic equilibrium of the drum membrane and produce minute solutions of continuity in the dermal layer, thereby allowing cocain or its succedanea to reach the nerve filaments, are the best we have at present for use in the external canal.

The conditions favoring this application of cocain are: (1) The removal of foreign substances and loose scales from the drum membrane and canal. (2) Dehydration of the outer layers of the membrane—a desiccation which causes molecular contraction and interstices through which the anesthetic can reach the deeper parts and nerve terminations. (3) The induction of endosmosis. The first condition is met by the use of hydrozone which is stronger and better than any other kind of $H_2O_2$ preparation in softening and boiling out the débris of the canal and in lessening the resistance of the dermal layer. The hydrozone is subsequently mopped out by cotton applicators or syringed from the canal. The second and third conditions are met by the use of alcohol and aniline oil. The latter is absorbed more slowly and its effects last longer than the former. The solutions used are 5 to 20 per cent. of cocain in equal parts of absolute alcohol and aniline oil. Anesthesia is gained in 10–15 minutes. The disadvantage of the solution is that the aniline oil is toxic and obscures the field. The external canal is generally filled to ensure osmotic instability and certainty of penetration. The toxicity can in a great measure be prevented by not filling the canal but by applying to the drum membrane a small wad saturated with the solution and by making only one application. The obscuration of the field by the dark oil will then be less and the solution can be more easily mopped away.

For the last six years I have experimented desultorily with tubal injections of cocain to desensitize the drum membrane. I have tried fractional experiments, applying the anesthetic to the pharyngeal orifice, to the cartilaginous portion and to the deeper surface of the tube and to the drum cavity by means of a Weber-Liol catheter or a virgin silver modification. I have come to the conclusion that the Eustachian tube is the only channel through which local anesthesia can be best obtained.

In the embryo $\frac{7}{8}$ of an inch long, the drum membrane is represented by connective tissue, bounded below by the external canal which forms its skin covering and bounded above by the Eustachian tube which forms its mucous covering.

From this embryological formation and from the identity of nerve supply we find the reason for the fact that anesthesia of the deeper portions of the tube will produce anesthesia of the drum cavity aud membrane. It may seem like begging the question to state this, but my trials have forced this home to my mind. I do not believe that the 5 or 6 minims I blow into the tube are sprayed by the Politzer bag into the tympanic cavity. I think that absorption of the cocain by the tubal mucous membrane affects the drum and membrane intermediately and by reason of continuity of structure. The fact that cocain anesthesia has a field of action of about an inch from the spot to which it is applied would likewise bring the tympanic membrane within the area of tubal anesthetization.

Unfortunately the lymphatic system of the ear is not well known. If I may be allowed to digress, I think that the production of acute otitis media might be explained more by the theory of absorption from a tubal focus or of continuity of structure than by the mechanical one (sometimes urged) of septic matter blown through the tube into the tympanic cavity.

After having forced the cocain solution into the tube, I have found that in a short time—a time varying in length according to the amount of vascularity present—probing the different areas of the dermal surface of the membrane would occasion little or no distress.

My observations with this method of comparative sensibility do not coincide with those of Dr. Blake, who finds that the areas of the membrane from below upwards and from the umbo backwards increase in movement, vascularity and pain. I have sometimes found a trifle of sensibility at the lower margin of the membrane, and at the region of the stapes entire absence of any but tactile sensation.

These facts and observations on atrophic drums have shown me that the dermal layer need not be considered in local anesthesia of the membrane and does not play so great a part in sensation as the mucous layer, since palpation of the skin surface does not elicit pain although it reaches only the mucous membrane. 2d. That the pain in palpation does not result from the local impact but from the excitation of the whole sensory apparatus of the tympanic cavity, induced no doubt by the sudden abnormal inward movement of the drug contents. 3d. That the pain of incision depends on the pressure made on the drum membrane by the knife as much as on the cutting. 4th. That the incision should consequently be made with the minimum of inward pressure and with as sharp and as thin a knife as practicable. This explains why incision in the membrane is made so much easier by the use of the Græfe knife than by the poor knives made especially for the work—knives whose smallness of blade precludes sharpness of edge. 5th. That in order to produce the best results in this method of anesthesia isotonic or iso-osmotic solutions of cocain should be used in order to avoid edematization of the tube and subsequent transient otitis media.—*New England Medical Monthly, November Issue.*

---

### THE PROPHYLAXIS OF VARIOLA.

BY C. A. CORDINER, B.Sc., M.D., CITY PHYSICIAN TO ASTORIA, OREGON.

In order that an outbreak of smallpox may take place two factors are necessary: (1) a number of susceptible individuals; (2) the introduction into the body of the virus upon which the disease depends.

Now, the first is easily obtainable, due to the lax laws in many communities governing this protection, and the virus may come from any one of several sources.

(1) Persons already sick with the disease.

(2) Persons not themselves sick, or susceptible, but who having come in contact with the poison, may convey it.

(3) Fomites (old cotton or woolen).

(4) The bodies of those dead with smallpox.

(5) The air surrounding a patient with the disease. It is not known to what distance the virus may be wafted through the air, but the distance as quoted by some men seems to be extreme. I do not believe it can be transferred more than a few yards.

For the prevention of the spread of smallpox different practices have been in vogue, namely inoculation, vaccination, disinfection, isolation.

Inoculation was introduced about the end of the seventeenth century, and was practiced among some daring individuals. It was not destined to continue long as a custom, nor did it have a large following in Europe, for it simply meant the inoculation of a person with the virus of smallpox itself. I can compare it with nothing more than a man who will set fire to his house, when he becomes aware that the block in which he lives seems doomed by that element. It was a decidedly poor practice, for not only was it quite possible that the people who were inoculated, thus making them sure of an attack, might have escaped, but persons with whom they came in contact might also have escaped infection, and I do not see how the virulence could be lessened at all, as it simply meant the passing on of the disease from one to the other.

Vaccination, on the other hand, gives us something different; it means the introduction into the system of something which we know to be milder or will antagonize that more dreaded disease. In what way it does this is not definitely settled; whether it leaves the elements behind detrimental to the welfare and growth of smallpox, or whether it removes from the economy elements common to their growth, is not for me to prove. In whatever way it does its work, it is most gratifying to know that we possess it, and we can never praise too highly that grand old country doctor, Edward Jenner, for his great work. The work by him entitled, "An Enquiry into the causes and Effects of the Variola Vaccine, etc" was one of the greatest blessings ever conferred upon mankind.

Of course, some things can be said against vaccination, for example, the unsightly scar, the swollen arm or leg, etc., but to what is this due? Frequently to the careless doctor himself, but much more frequently to the careless, dirty patient.

I vaccinated one boy, three years old, last January. I first cleansed the surface with boiled water, then with ether, rubbing dry with sterilized gauze. With a surgically cleaned needle I scraped the outer layers of the skin and with a capillary tube, prepared in the proper manner, I inoculated the boy; after letting it dry for a few minutes, I applied a shield and sent the boy home with his mother, who, during the operation was quite lavish in her caresses on the lad, with instructions to return in a few days if the arm showed any signs of becoming bad. Well, ten days after, that boy returned, and with what. Well he had an arm and an axillary abscess or two, and a horribly dirty bandage. I asked the mother what she had done. She looked angry, and proudly said that "*that thing*" which I put on his arm itched so that she tore it off and put some vaseline on it and a bandage.

Think to how many uses that vaseline may have been put to, where every one comes along and dips his fingers into any corner of the bottle, and the bandage was not sterilized—far from it. Well, I then had on my hands what only the many of us who practice vaccination have. We are not to blame, and I cannot see where the vaccine is to blame for such results. I could cite other and even more absurd cases than this, where people will date malignant or severe gastric trouble from their vaccination, when it is only an unfortunate coincidence.

May the day be hastened when the medical world, as well as the laity, will recognize the true protection of vaccination. Of course, the opposition will say we have smallpox just the same. Why certainly we do; we know that, but with what results? Do our statistics show anything as we compare the mortality of the vaccinated as against the unvaccinated? What can be more conclusive than the mortality from smallpox in Cuba since our flag came to that shore? Why, before vaccination was made general the death-rate was six times as great as now, when vaccination is enforced. This is not the only instance on record, as death-rates from its introduction will show. But will not some of the cases have smallpox twice? Surely they will. And how are we to account for it? In about the same way we know that the introduction of vaccine virus will prevent the catching of smallpox in a great many cases, and render it a very much less dangerous disease, if not harmless, in the others.

Many of our opponents will argue against it, in that we may inoculate other diseases, such as syphilis, at the same time. Well, we may, if we live so "away back" that we cannot get a nice sterilized glycerine tube, and will then take a scab off some young buck's arm and, after maceration, introduce it into the arm of others, little wonder that such unfortunate events take place. Does that speak badly for vaccination? Not at all. It is either the unwise selection of the operator or his mercenary disposition which prevents him from buying glycerine tubes or ivory points. I have not much choice between these. Of course, much could be said on both sides, but the small tubes, with their sterile glycerinized contents have a little the advantage in my affections. Certainly, most of us have met with disagreeable cases—ones that would almost cause you to say you would never vaccinate another, but most belong to the class I have quoted and the remainder to a class not less ridiculous.

And now I shall consider isolation. This is something all agree on, and it cannot be carried out too rigidly. The moment a patient is suspected he should be taken to a suitable dwelling, far enough from any public interference, and a nurse placed over the case—that nurse to be with the case all the time. The attending physician should be provided with a pair of rubber boots, a rubber coat and a hat with a mask entirely encircling his head, leaving only small holes for his eyes and to breathe through; likewise rubber gloves. It is not necessary for him to come in contact with the patient himself if he has an intelligent nurse, and if he does not actually inhale the patient's breath, and removes the rubber clothing on leaving the abode, and then sterilizies the face and hand with some antiseptic wash, there is little danger of his either contracting the disease or bringing it to others.

Disinfection would be unnecessary if we did not have any cases, but unfortunately we have, and until we find something equally as or even more effective than vaccine to prevent it altogether, then we must use it. The clothing used by the patient and nurse during the sickness should be destroyed, the room or rooms occupied or visited by either should be closed tightly and sulphur or some other disinfecting material burned and the walls of the room thoroughly scrubbed with some antiseptic, such as corrosive. After the patient has left the quarters he should be obliged to bathe thoroughly and dress in new clothes. He is now fit to enter into the world again, little the worse for his attack, and with few pits, if all cases were as light as those occurring in my neighborhood during the last year.

I will say that out of sixteen cases which we had in and about our city, we had no deaths and but one malignant case. That case has not been vaccinated. Each new case came from other towns and was not contracted here.—*Medical Sentinel.*

---

### URTICARIA, ITS VAGARIES AND TREATMENT.

This cutaneous eruption in its different forms is so often an accompaniment of other diseases and morbid conditions of the system, that it would be more properly designated as a symptom than as a separate disease. We find it generally described as a non-contagious inflammation of the skin attended by the development of ephemeral round or oval wheals, either red or whitish in the center, with a surrounding red areola. These elevations range in size from a small papule to nodules of an inch or more in diameter and even sometimes running into edematous infiltrations of considerable extent. The prominent subjective symptoms are itching, burning and tingling sensations. This eruption is sometimes attended by a high febrile movement and serious constitutional disturbances, at other times the general organism seems to be in a fairly normal condition. During the course of many contagious and visceral diseases, urticaria is an incidental feature which often embarrasses the diagnostician.

The antitoxins, new drugs and numerous pharmaceutical preparations which are being constantly prescribed by physicians, make these urticarial eruptions much more common than they formerly were. The extravagant habits and luxurious indulgences of modern life, with their resulting dyspeptic and neurotic disorders, have also contributed much to the increasing prevalence of urticarial eruptions. Imperfect transformation of tissues and retention in the system of toxic material which should be excreted, add their influence to the long list of causes which provoke these cutaneous disorders. But all these things alone would not be sufficient to produce urticarial eruptions without the existence of a predisposition to the occurrence of serious exudation of the cutaneous tissues, from the fact innumerable internal and external causes which are constantly operating upon a large class of people, do not in very many cases provoke urticaria. A peculiar condition of the skin has been revealed in certain susceptible persons by what is called dermographism. With a pencil, names or figures may be written on their backs; these names or figures are simply linear infiltrations produced by the movement of the pencil on the person's skin. Urticaria is undoubtedly the expression of a vaso-motor disturbance of the skin tissues in persons of irritable neurotic proclivities.

The poison of smallpox will sometimes produce a prodromal rash in the form of an urticaria a day or two before the development of the typical smallpox eruption. This happens only in that class who have urticarial susceptibilities. The toxic products of undigested food absorbed and thrown into the circulation act as irritating substances and provoke urticaria in those predisposed to such lesions of the skin. The mucous membranes, or the internal skin, may also develop these lesions; and possibly in the serious febrile cases, where there are gastric and intestinal complications, these internal lesions are responsible. When the urine shows a marked decrease in urea and uric acid, often the most stubborn cases of urticaria result. I have known these cases to be promptly relieved by the administration of colchicum or salicylate of soda, when all other remedies had failed. In all chronic and apparently incurable cases the urine should be tested, when it will often be found that there is a deficiency of organic salts, with their probable retention in the system.

In some persons with peculiar idiosyncrasies, urticaria results from the ingestion of certain kinds of food. Certain kinds of fish, meat, vegetables and fruit will provoke an attack. In many of these cases it is very likely these articles of food have undergone some putrefactive changes before they were eaten, evolving toxic products which excited the trouble. Most people know little and care less about the quality of their food as long as it is palatable. The same is true of the beverages they use, and for this very reason the physician should give such matters his special attention. The success in treatment of all skin diseases is achieved more signally along dietary, than medicinal lines. This is often demonstrated in patients who, after having been for months drugged without any benefit by one physician, are cured by another who prescribes hygiene and diet, instead of drugs.

In most cases of urticaria, saline aperients, diuretics and intestinal antiseptics with rational hygienic and dietary management will soon effect a cure. When these means fail, a searching effort must be made to ascertain and remove the cause. Let attention be directed to the correction of those inherited or acquired nervous susceptibilities which operate as predisposing causes. Build up crippled organs and restore functional activities. Cleanse the stomach and intestinal canal, and keep them free from ptomaines and other toxins. See that the stomach and intestines furnish the proper secretions for the purposes of digestion, and that the liver and pancreas supply the proper digestive ferments to prepare the food for absorption and utilization. See that the kidneys, liver, lungs, skin and bowels remove from the system all waste material and obnoxious matter. Attention to all these details in some cases is absolutely necessary to insure successful results.

During convalescence, if the vital powers have been reduced, tonics, with alkalies and acids, according to the requirements of the case, may be prescribed. In some cases of an intermittent form, quinine should be given, and when rheumatism is associated with urticaria, a combination of iodide of potassium, bromide of potassium and chloral hydrate, each in ten-grain doses, may be prescribed three or four times a day. Atropine in doses of one one-hundred and twentieth of a grain three times a day will sometimes be serviceable. Salicylate of soda in fifteen-grain doses in obstinate cases, three times a day, will often give prompt relief, but smaller doses will rarely be of any service. Iron, arsenic, and strychnine, in connection with saline aperients, in some chronic cases, have a beneficial effect. Warm and cold baths alternated may at times be used to good effect. The excessive itching will often yield best to the following lotion: Bichloride of mercury grs. x, spirits of rosemary and alcohol each ℥i, emulsion of bitter almond ℥vi. M. Sig.—Sponge the parts two or three times a day.—*St. Louis Medical Era.*

---

### MEDICAL EDUCATION IN THE UNITED STATES.

Medical education in the United States has been the derision of all European countries from time immemorial. The reason of this is that too little care has been given in the requirements of admission to our better institutions, and in some States colleges have been allowed to flourish that were mere diploma factories for the enrichment of private individuals.

Happily both these conditions are better to-day; on the one hand the requirements for admission in most States are more exacting, and good legislation has made it impossible for any one to purchase the degree of doctor of medicine. Besides this, in most States an examination for a license to practice is required after the candidate has received his doctor's degree.

The majority of our better universities require a collegiate degree for admission or an equivalent examination which satisfies the faculty as to the ability of the student to be benefited by its instruction.

In the State of New York this is regulated by the Regents at Albany, who also control the examination for the right to practice after graduation.

The course in medicine is limited to four years, which is supplemented by one or two years of hospital training; the latter is not obligatory, but is strongly recommended and usually followed.

In England the requirements for admission are somewhat different; there the course in medicine contains certain branches, like botany, geology, biology, chemistry and physics, that are usually taught in the undergraduate department of our colleges, and which course leads up to the degree of bachelor of science with us, while with them after three years' study in medicine, the successful candidate, after examination, receives the degree of bachelor of medicine or surgery, according to the particular course of instruction pursued. It contains a little more science and some elementary instruction in medicine, but is minus the mathematics, philosophy and languages that our college course contains, the degree from which is taken here as our standard of admission.

From this point on the courses of instruction are about parallel, consisting of didactic lectures on practice of medicine, therapeutics, anatomy, physiology, obstetrics, gynecology, surgery, pathology and hygiene, together with laboratoay work, dissection, clinical lectures at the hospitals, and ward classes of a limited number of men at a time.

In England every man must have delivered more cases in obstetrics than is required here, and many of the things, such as vaccination, he is required to practice on the patient before graduation, which he gets in his postgraduate work in the hospital here.

The course in surgery is very thorough in England, and is held separate from internal medicine, but the requirements to perform operations on the dead body besides passing an oral examination hold as well here as there.

The making of autopsies and the studies in gross morbid anatomy and pathologic histology are held equally important by both countries.

Study in the special branches, such as the eye, ear, nose, throat and skin, is carried on at the dispensaries and hospitals, and an examination is required in these specialties in our leading universities as well as it is in England.

The clinical material of the hospitals in England is abundant, perhaps more so than here, and there are greater facilities for individual study of cases than there is here, but this is a question that will be arranged satisfactorily in America just as soon as the universities get greater jurisdiction in the hospitals.

England has one great diploma that gives a doctor the right to practice anywhere in the British Empire, and that is the certificate of the Royal College of Physicians and Surgeons. To gain this one must already hold the degree of doctor of medicine, and pass a thorough examination before an exacting examining board of the college before he can become a fellow.

Much has been said about establishing a Federal College of a similar nature here in America at Washington, where physicians could obtain a certificate, after thorough examination, that would permit them to practice anywhere in the United States.

As the laws of the different States now stand, in most cases it is necessary for a doctor who has the right to practice in one State to pass another examination in the State where he proposes to remove before he can take out a license to practice there.

All things taken together great strides have been made in medical instruction in the United States in the last quarter of the century. Many of our students go abroad for supplementary study after graduation, and they have brought home new ideas, instruments and methods that have given impetus to our progress toward a higher standard, so that to-day we have universities here that give courses, under the directorship of men who are known and respected abroad, that compare in scope and fulfillment with those of any European country.

We can also be proud of many of our medical achievements here in America; especially noteworthy among them are the works of Beaumont in gastric digestion, the studies of typhus and typhoid fevers by Gerhard that led to a positive differentiation, the recognition of the contagiousness of puerperal fever by Holmes, the work of Wood in therapeutics, the planning and carrying out of the rest cure by S. Weir Mitchell, the performance of the first laparotomy by Ephraim McDowell, and last, but not least, the discovery of the anesthetic power of ether by Morton.—*Gaillard's Medical Journal.*

---

### *AN ORIGINAL METHOD OF SETTING AND TREATING A FRACTURED KNEE-CAP.

In a brief way, I wish to call attention to a method of setting and treating a fractured knee-cap devised in an emergency, which, for simplicity, safety and effectiveness, far surpasses any other method to which my notice has been called.

The apparatus consists of a flexible rubber ring, three pieces of adhesive plaster and a bandage.

I hold in my hand a ring formed by a spiral spring, covered with rubber. We are all familiar with it as a rubber ring pessary. It can be procured at almost any drug store, of different sizes, so as to fit any knee-cap.

Its application is simple. The patient is put to bed; the knee is placed in extreme extension, then the fragments—be they two or more—are brought together. When the greatest degree of approximation possible is obtained, the ring is slipped over the projecting patella. The ring will cosily keep the fragments in position so long as it is held in place, which is accomplished by

---

* Read by R. Bruce James, M.D., Danville, Va., one of the Medical and Surgical Staff to the Home for the Sick, Danville, before the Thirty-third Annual Session of the Medical Society of Virginia, held at Newport News, Va., September 23-25, 1902.

attaching two strips of bandage or rubber adhesive plaster to the sides of the ring and carrying these around the joint and fastening in rear. A sufficient degree of tension should be exerted on these strips so as to insure the position of the ring. Two other strips of plaster should be fastened to the thigh and leg—passing diagonally over the ring. A light "figure of 8 bandage" over all completes the operation. The limb is placed at an elevation by means of a board that extends from the hip-joint to the foot-board of the bed. Moderate cold is applied by means of ice-bags. This position is maintained four to six days—till all spasmodic contraction of the quadriceps ceases, and the parts are free from swelling and pain. Then with a light rear splint, added for safety, the patient can go about on crutches, or sit in a chair with his leg extended. The "figure of 8 bandage" can be removed at any time, without molesting the fracture, and thus you can, at a moment's notice ascertain the condition of affairs. The ring soon makes for itself a snug bed around the injured bone which it holds close in its embraces, and the surgeon realizes that there is little cause for uneasiness as to the proper coaptation being maintained. By thorough cleaning of the skin and ring with soap, water and alcohol, before using, there is no danger of excoriation.

I want to report two cases:

*Case I* was a dancing master, whom, some years ago, I was called to treat. I found fractured patella. While casting about for the best thing to do, I caught sight of a ring pessary in my satchel. I brought the two fragments together, and slipped the ring on. It fitted snugly and held the parts together perfectly. I fastened it as above indicated. In one month the patient was walking with only a slight limp. In two months he was giving dancing lessons again. I saw him six months later, when he had perfect use of the limb.

*Case II.*—During the past winter I was called in consultation to see a case of fractured knee-cap eighteen days after the accident. The fracture had been set on the day of the accident by a competent surgeon, who employed the usual method recommended by Wyeth and others. I found the two fragments had slipped over an inch apart. These fragments were brought together as nearly as possible, but they lacked about a quarter of an inch of complete approximation, when the ring was put in position and secured as already described. This degree of approximation was well maintained until recovery, which was somewhat delayed, owing to the fact that ligamentous union had begun before the fragments were brought together. The patient wore the ring for two or three months as a precaution, but ultimately made a complete recovery and to-day he has a normally useful limb.

On looking up the literature on this subject, I find allusions made to the fact that an iron ring had been used for the purpose of coaptation, but no one appears to recommend its use. There are obvious defects in and objections to the iron ring which do not obtain in the use of the soft, flexible instrument.

The advantages of the methods I have described are :

1. It holds the fragments of fractured bone together perfectly.

2. It is simple and easy of application.

3. It permits easy inspection without molesting the fracture.

4. The lightness of dressing permits application of heat or cold whenever needed, or as long as desired.

5. The short time it is necessary for the patient to remain in bed.

6. Last, but by no means least, it is perfectly safe and effectual. — *Virginia Medical Semi-Monthly.*

---

### THE ACCIDENTS OF ANESTHESIA: THEIR PREVENTION AND TREATMENT.

Eisendrath (*Am. Med.*, Nov. 15, 1902), in a well-written paper on this subject, after enumerating the various agents used for the induction of anesthesia, makes a comparison of their physiologic action. While ether increases arterial tension and stimulates the heart, chloroform decreases arterial tension and depresses the heart. He lays down the following rules for the choice of an anesthetic: 1. Ether should not be given when there is an increased arterial tension and blood pressure, for example in atheromatous conditions. 2. It should not be given when there is great tympanites or under conditions which will interfere with action of the diaphragm.

3. It should not be given to nephritic patients. 4. It should not be given when the postoperative treatment requires a prolonged recumbent position, for as I shall show later the hypostatic congestion of the lungs plus the increased amount of salivary secretion, which latter ether causes, favor the development of pneumonia. 5. Ether is contraindicated in individuals who have from any cause a hyperemic condition of the respiratory tract. The increased secretions which it causes are so frequently aspirated into the lungs. Contraindication to the use of chloroform is in any condition in which there is a decreased blood-pressure, owing either to great loss of blood or some systemic disease, for under these conditions the tendency of chloroform to increase the already diminished blood-pressure will cause a rapidly fatal syncope. 6. It is contraindicated in affections of thc heart in which there is lack of compensation, in myocarditis and in pericardial adhesions. Have had occasion to administer chloroform to a number of patients with compensated valvular affections and could not observe any unpleasant symptoms. At a recent meeting of the Paris Medical Society the general conclusion was that there was no objection to administering chloroform to patients with heart disease provided there was compensation and no myocarditis. 7. Chloroform should not be administered in the so-called status thymicus or the thyroid state. 8. Chloroform should not be given in either acute or chronic septic conditions, the heart being already weakened through the action of toxins. 9. Chloroform should never be given when it is necessary to administer it for more than an hour, on account of its degenerative effect upon the heart muscle and parenchyma of the liver and kidney.

He summarises as follows:

1. Chloroform has a narrower zone of safety than ether. Its toxic effects are as a rule manifest at the time of administration. Ether is the cause of death in many cases through renal or pulmonary complications from hours to days after the anesthesia. The late deaths due to chloroform are so rare as to render this factor practically of no importance. Chloroform is a more dangerous anesthetic than ether and must be watched far more carefully. 2. Chloroform kills more frequently through primary cardiac than respiratory syncope, and the anesthetizer must watch constantly the degree in volume and rapidity of the pulse, indicating the fall of blood-pressure, and a slowing of the more shallow respiration. Chloroform syncope can be avoided by keeping the head low, if possible turned to one side, keeping the jaw forward, watching the pulse, respiration and pupil, keeping the patient's mind quiet, and keeping the chloroform well diluted with air. 3. Ether rarely causes death through its immediate, but more frequently through its after-effects, such as pnuemonia and uremia. These complications may be avoided by keeping the head lower than the level of the body, turned to one side, and not giving the ether in too concentrated a form; also by not keeping the patient on his back too long, and by relieving post-operative tympanites as soon as possible. The contraindications to the use of chloroform are myocarditis, pericardial adhesion, and uncompensated valvular disease. In all other forms of heart disease it may be given. It should not be given when the blood-pressure is low or in status thymicus, or when a prolonged anesthesia is necessary. 4. The pulmonary complications are relatively more frequent with local anesthesia than if a general anesthetic is given. They may be due to aspiration of mucus or food, or due to hypostasis and to embolism. The latter is far more frequent than is ordinarily thought. Avoid these by exposing patients as little as possible. Use heated operating tables, avoiding recumbent position and tympany. 5. Avoid renal complications by careful examination of the urine before anesthesia. 6. Begin process of resuscitation immediately and systematically: Artificial respiration, the method of König-Maas, or massage of the heart, rhythmic traction of the tongue, method of Prus, or direct exposure of the heart, and intravenous salt transfusion. I prefer to begin with massage of the heart and rhythmic traction of the tongue (16 to 18 times a minute.

---

### MRS. EDDY'S AMENDMENT.

Following the death of a child at White Plains, New York, from diphtheria, under Christian Science treatment, a wave of popular resentment against the cult has swept over the country.

It has stimulated Mrs. Eddy to promulgate through the official organ of the church, the Sentinel, an order that "until the public thought becomes better acquainted with Christian Science, the scientists shall decline to doctor infectious or contageous diseases."

In the editorial Mrs. Eddy further says:

"On the subject of reporting contagion, I have this to say:

"I have always believed that Christian Scientists should be law-abiding, and, actuated by this conviction, I authorized the following statement about one year ago:

"Rather than quarrel over vaccination, I recommend that if the law demand an individual to submit to its process, he obey the law and then appeal to the Gospel, to save him from any bad results. Whatever changes belong to this century, or any epoch, we may safely submit to the providence of God, to common justice, individual rights and governmental usages.

"This statement should be so interpreted as to apply on the basis of Christian Science, to the reporting of contagion to the proper authorities, when the law so requires."

Now if this she-prophet will only go a step further and advise her adherents not to attempt the "treatment" of any actual pathologic lesion, but to confine their ministrations to those who are simply suffering from a non-material impediment to thought and a congenital absence of the reasoning faculty, all will subscribe to the revised creed.

Physicians have discussed this subject until it has grown flat, stale and unprofitable to them. In slang phrase it is "up to" the public.

William Marion Reedy, editor of the *St Louis Mirror*, who has previously spoken his mortal mind regarding Eddyism, says:

"The New York Board of Health reports the death of a little child from malignant diphtheria and Christian Science prayers. To questions why no physician had been called, the mother naively replied that 'we don't have illness, but have claims and errors, and if we properly pray, and trust in God, we will recover.' One of the praying healers hastened to add the statement that Christian Science heals 'through the power of God over the body through the mind.' A case and vaporings of this kind are enough to stagger the senses of anybody that is not confined within the walls of a ‘bug-house.’ Could there be anything more idiotic than Christian Science and its doctrines? And yet, there are many among the educated classes who unhesitatingly assert their utmost faith in the teachings and truth of this preposterous religious idiocrasy and fly into veritable fits of rage whenever the insinuation is made that Christian Science is nothing but a monstrous fake. The adherents of this bizarre cult are as fanatical as the mad Mullahs of Somaliland. And rightly so, for there is hardly any difference between their beliefs and those of the Mullahs and negroes of Africa. Du Chaillu, the African explorer, is authority for the statement that ‘the Camma theory of disease is that Okambo (the devil) has got into the sick man. Now this devil is only to be driven out with noise, and, accordingly, they surround the sick man and beat drums and kettles close to his head; fire off guns close to his ears; sing, shout and dance all they can. This lasts till the poor fellow dies or is better.’ In Dahomey, we are informed by Joseph Alexander Tillinghast, the priests of the natives cure diseases by driving out the evil spirit. These wild tribes of Darkest Africa believe that all disease is spiritual and should be healed by spiritual means. Christian Scientists believe about the same thing. If you suffer from a well-developed case of the mumps or appendicitis, there is nothing especial the matter with you, except that you are the victim of ‘error,’ and all you have to do to rectify the error, is to pray or have somebody else pray for you. There is no disease in the world—what is known as such is ‘error,’ or ‘claim.’ This Dahomeyan idea is wonderfully simple and primeval. It is as simple and primeval as the intelligence that brought it forth and that believes in it. To identify the teachings of the Saviour with such rot as these un-Christian Scientists are exuding is a stupendous impertinence. There is as much affinity between fundamental Christianity and Eddyism as there is between the writings of Thomas Aquinas and of Voltaire. Eddyism is Dahomeyanism and fit for those only who live in kraals and walk around in the altogether.”—*St. Louis Medical Review.*

### Ankylostomiasis, the Most Common of the Serious Diseases of the Southern Part of the United States.

By H. F. Harris, M.D., of Atlanta, Ga.

In *American Medicine* for July, 1902, I reported a case of ankylostomiasis. After my article had been sent to the publishers the distinguished helminthologist of the United States Bureau of Animal Industry, Dr. Charles Wardell Stiles, announced the important discovery that a species of *Ankylostimum* exists in this country that is not found in the old world, and differing in some important particulars from *Uncinaria duodenalis.* This new parasite he has named *Uncinaria americana.* It may as well be stated here that Dr. Stiles has kindly examined the parasites from one of my cases and has found that they belong to his new species; in all other instances where the worms were obtained the like was found to be true. The discovery of a distinct American species of the hookworm is very important, as it leads to the inference that the aborigines of this country were infested with this parasite, and that the worm is probably present in all parts of the United States where the conditions are suitable for its development.

My observations during the last six months bear out this assumption in a most striking manner. A few weeks after my first case of the disease was seen a second one was encountered that originated in middle Georgia, but though I was constantly on the search for it, no other instance of the disease was found among the numerous patients that came to the clinics of the Atlanta College of Physicians and Surgeons. In June of the present year I made a trip to north Georgia, a region that has long been noted as one in which the inhabitants are very pale and anemic, this condition being commonly reputed to be the result of dirt-eating. Here I saw many instances of what was in all probability ankylostomiasis, but as a result of the ignorance of the people and their suspicion of all strangers, a proper examination could be obtained in only four cases, in all of which the parasite was demonstrated. Subsequently a case of the disease was seen that originated in middle Alabama. During September and October I have been studying malaria in south Georgia and Florida, a region in which the people show profound anemia even more often than in north Georgia. This condition is commonly ascribed to malaria, *but my observations show that in almost all instances the sufferers have no malarial parasites in their blood, but eggs of the ankylostoma are constantly found in the feces.* During my entire stay in this region I only saw one case of profound anemia from malaria, and in this instance the patient did not exhibit the extraordinary anemia so commonly found in those infected with the ankylostoma. I feel no hesitation in saying that time will show that by far the greater number of cases of anemia in Georgia, Alabama and Florida are due not to malaria but to the ankylostoma, and that this is the most common of all the serious diseases in this region. There can be no reasonable doubt that what is true as regards the States named likewise holds good for the entire south. Since my first case was reported thirteen other instances of the disease have been seen—eleven originating in this State, and one each in Florida and Alabama, and if all of those encountered who were suffering from anemia could have been examined there can be no doubt that the number would be manyfold greater.

This communication is written in the hope that southern physicians will take up this most important matter at once, for in no other serious disease does the victim suffer so long, in no other condition is he for such a period a menace to those about him, and in no other malady of such gravity is the treatment so rapidly and surely successful.

I shall be very glad to report without charge on specimens of feces sent me for examination. A very small quantity of fecal matter is sufficent for this examination, but it should be sent in a bottle in order to keep it moist.—*American Medicine.*

---

### PRESIDENT DAVIS'S ADDRESS.

President Davis in his address said the organization grew out of the opposition to the Alabama Surgical and Gynecological Association of State Health Officer Jerome Cochrane. Dr. Davis, the prime mover in the organization of the Alabama society, intended that similar associations should be formed in the different States, which should be the constituent parties to a southern association to be formed at a later period. The opposition of Dr. Cochrane hurried the movement and caused the enlargement of the Alabama association into the Southern Surgical and Gynecological Society. The society has a number of honorary members, and the president recommended that provision be made for life members, and that surgeons of great eminence who are more than sixty-five years of age, or who have been members more than twenty years and attended more than 50 per cent. of the meetings, be entitled to this membership. He suggested for such membership Dr. J. McF. Gaston, Atlanta, Ga.; Dr. R. B. Maury, Memphis, Tenn., and Dr. Thaddeus A. Reamy, of this city.

He advised the association to limit its membership to 200, and urged that the secretary's salary be increased to $500 a year.

He said that the special societies had wielded a wonderful influence in the medical profession, and that medical literature had been enhanced in every way by them, and as individual societies they should be encouraged, but their union into a national congress he did not think would be conducive to the best interests of the medical profession.

"The special societies, composed as they are of the leaders in the several specialties," he said, "are under obligations to the American profession to assist in the better organization of these sections. Many of the members of these societies are officers and active working members of the American Medical Association, and can accomplish this result. There must be one class of membership for the section that can be held by only those who are recognized as teachers and leaders in order to make membership very desirable and sought after. I would suggest that this class be known as Fellows, and that they pay in addition to the annual dues of the association $5 annually for section dues, which fund would be used in the publication of the proceedings of the section. The officers and authors of papers should come from the Fellows. All members would have the privilege of taking part in the discussion, and thus an opportunity would be provided for the association members to show themselves worthy of a membership.

He stated that the south had suffered greatly from the removal to the larger cities of the east and north of many of its ablest surgeons and gynecologists. This was due largely to the poor fees received by southern surgeons. With the south's great increase in wealth he did not think this condition should obtain in the future.

He paid a tribute to the medical profession of the south, and spoke of McDowell, Sims and Battey as epoch-makers in surgery. He also referred in glowing terms to the work of Dudley, Paul F. Eve, Warren Stone, Dugas and Pope. Of the many now dead who had done excellent work in recent years, he mentioned the names of McGuire, Yandell, Briggs, Rogers, Sr., Kinloch, Westmoreland, Sr., Campbell, Gilmore, Nott, Mastin and Richardson.

He said the American profession had always been foremost in all that was good for the country, and after the civil war it was prominent in its efforts to break down the barriers between the sections. Said he: "It was Dr. W. O. Baldwin, of Alabama, who, in 1868, when the physicians of the south were invited to reunite with the American Medical Association, advised them to accept the invitation, and at the next meeting in Washington he was elected president."

---

### THE VALUE OF GUDE'S PEPTO MANGAN IN ANEMIA.*

Anemia is a very common disease in this country (Cuba), and consequently one against which the physician is often obliged to contend in the practice of his art. While the use of the ordinary iron preparations often give all the effects that could be desired, yet it usually produces a condition which may be regarded as a secondary disease—constipation. In looking about for a preparation which would not present this very serious disadvantage, which cannot always be counteracted by the coincident administration of laxatives, we came across Gude's Pepto-Mangan, which, according to the published statements of many clinicians, seemed to us a remedy worth trial in a large series of cases. Accordingly, we obtained a sufficient supply of this preparation for our hospital,

*By Dr. Enrique Diago, Havana, Superintendent of Hospital No. 1, Havana, Cuba, and Dr. José F. Benitez, Havana, Chief of the Laboratory, Hospital No. 1, Havana, Cuba, Translated from "Progreso Medico," Havana, April, 1902.

and began to treat all our cases of anemia, in which iron was indicated, with Gude's Pepto-Mangan.

In presenting now the results of our observations with this pharmaceutical compound, we may say at once that our expectations were more than realized, when we noticed its efficiency in combating the disease, and its perfect palatability and freedom from constipating after-effects.

One of us, Dr. Benitez, chief of the laboratory of the hospital, undertook the task of keeping minute records of all the cases observed, including a record of the amount of hemoglobin and of the number of the red blood-cells, both before and after the treatment. For the purpose of illustration, we relate briefly six cases, which show conclusively the effects of Gude's Pepto-Mangan on persons with anemia, and prove without doubt that the administration of this remedy is connected with none of the disadvantages and discomforts attending the use of the ordinary preparations of iron.

*Case I.*—N. G., aged twenty-six years, was admitted to the hospital, suffering from loss of nutrition, emaciation, pallor of the skin and mucous membranes, loss of memory, anorexia, mental depression,—in a word, from all the typical symptoms of anemia. This condition was traced in his case to a chronic malaria, from which the patient had been suffering for a long time. The patient weighed only 102 pounds at the time of admission.

Pepto-Mangan (Gude) was given in doses of two tablespoonfuls twice daily, at breakfast and at dinner respectively, with some cinchona wine. The first blood examination showed 2,400,000 red blood-corpuscles c.m., by the Thoma-Zeiss method. Ten days after the begiuning of the treatment, this patient, who had been so extremely pale when he entered, began to improve as regards the color of his cheeks and general appearance. His general well-being was so marked that he spoke with pleasure of the marked improvement in his condition which had taken place since he had been taking the new remedy at our hospital. In these ten days he had gained five pounds in weight and was able to walk around the ward without the lassitude which he had felt when he was admitted. The blood was examined a second time, showing an increase of 300,-

000 red blood-cells. The patient was discharged cured after fifty days' treatment, weighing 130 pounds and with a blood-count indicating 2,800,000 red blood-cells c.m.

CASE II.—Mrs. C. D., aged thirty-four years, who gave a history of miscarriage, was admitted with the symptoms of anemia, secondary to the loss of blood occasioned by the accident mentioned. The chief symptoms were emaciation, loss of strength, and gastro-intestinal disturbances. She weighed only ninety pounds when she entered the hospital, and her blood showed a marked diminution in the amount of hemoglobin, and only 2,300,000 red blood-cells to the cubic millimeter.

Gude's Pepto-Mangan was prescribed in the same doses as in the preceding case, and all went well until the tenth day, when the patient of her own accord, in order to facilitate the cure, and to accelerate the recovery, took five tablespoonfuls of the preparation during the day, causing a slight disorder of the stomach. The administration of Pepto-Mangan was thereupon discontinued, and tablets of bismuth and salol, together with a purgative were given. Five days later, the Pepto-Mangan was resumed, at first in doses of two teaspoonfuls, and two days later in doses of two tablespoonfuls. The further course of the treatment went on without any mishap, and the patient recovered completely. On leaving the hospital the hemoglobin was found normal, and the number of red blood-cells was found to have increased to 3,500,000 c.m., while the patient's weight had increased twenty-one pounds within fifty days.

CASE III.—Mr. M. D., aged twenty-six years, who had suffered during the preceding month from an attack of acute articular rheumatism involving a number of joints, entered the hospital complaining of the symptoms of anemia. He had the appearance of a convalescent, with pale skin and mucous membranes, fatigue in walking, emaciation, etc. There was edema about the ankles, but no valvular lesion in the heart, and there were in addition, absence of appetite, insomnia, functional depression of the genital apparatus, and dyspepsia. The patient weighed only ninety-two pounds, and his blood when examined showed a decrease in the amount of hemoglobin and only 2,500,000 red blood-cells c.m.

At the end of fifteen days' treatment, which consisted of the administration of two tablespoonfuls of Pepto-Mangan (Gude) at breakfast, of the same amount at dinner and of an additional tablespoonful at noon, the patient had gained a great deal of strength, his pallor had almost disappeared, the hemoglobin had increased and reached its normal quantity, and the red blood-cells had increased to 3,200,000 c.m. The patient was therefore discharged completely cured at the end of forty days after admission.

CASE IV.—Mr. R. G., aged forty-two years, who did not show any signs of organic disease, and who presented no characteristics of a gouty or lithemic diathesis, was admitted to the hospital in a greatly disturbed state of mind on account of attacks of vertigo, palpitation of the heart, extreme weakness, and various erratic pains in the muscles. He gave a history of a recent attack of influenza, during which his nervous symptoms had become intensified. He had not had a very marked rise of temperature, and the respiratory passages were scarcely affected during this attack, but there were severe pains in the back and joints, and an intense headache. The examination of the blood showed the presence of 3,000,000 red blood-cells c.m., and the patient was found to weigh only 110 pounds.

He was placed exclusively on Pepto-Mangan (Gude) treatment. Twenty days later, the pains had ceased; he ate well; his weight had increased to the extent of four pounds, and the red blood-corpuscles had increased in number by 200,000. Thirty days after admission he was discharged cured.

CASE V.—Miss C. P., aged sixteen years, was admitted to the hospital with a very pale skin and a deficient muscular and adipose development. Her menstruation had become irregular, and she had suffered from various nervous disturbances. Her growth had not kept in harmony with her nutrition, and she presented the characteristics of chloro-anemia, as frequently seen in Cuban girls—namely, accompanied by a series of neurasthenic symptoms. She weighed only eighty-seven pounds, and the blood-count showed only 1,800,000 red blood-corpuscles c.m. After ten days' treatment, the number of red blood-corpuscles increased by 200,000, and the weight of the patient by three pounds. Twenty-six days after admission she was removed from the hospital by her relatives, and on discharge her weight was ninety-four pounds.

CASE VI.—Mr. G. F., aged thirty-eight, whose previous history was negative, and who had not suffered from any severe illness shortly before admission, entered complaining of loss of flesh and strength, decrease of normal weight and extraordinary fatigue after his usual work. He attributed these symptoms to transgressions of hygienic rules. The first blood examination showed 2,600,000 red blood-cells c.m. The patient weighed 106 pounds on admission. Thirty-six days later, after having been under treatment with Pepto-Mangan (Gude) during the entire period, he was discharged at his own request. He had increased eleven pounds in weight and his red corpuscles numbered 2,850,000 c.m. (an increase of 250,000). He went back to his usual work without experiencing any unusual fatigue.

To sum up the results obtained with the employment of Pepto-Mangan (Gude) in the treatment of anemias, we may say conscientiously, that it is the best remedy we know of for this purpose, and that we do not hesitate to commend it to the medical profession at large, and especially to our confrères in Cuba, as an iron preparation that possesses all the advantages that can be demanded of such a remedy and none of the disadvantages that are characistic of other iron preparations. We would especially emphasize also that Pepto-Mangan (Gude) is very pleasant to the taste, and is most easily taken by patients of all ages and with the most delicate digestions.

Havana, March, 1902.

---

### SEMINAL WEAKNESS.*

Seminal weakness is known by several names, viz.: Seminal emissions, seminal incontinence, seminal leaks, true and false spermatorrhea. Recent medical and surgical writers are inclined to subordinate this disease. There are, however, honorable exceptions. Says one writer, "The most common derangements of the sexual

*Read before the Marion County Medical Society October 28, 1902, by James H. Brill, M.D., Indianapolis.

organs of man is a weeping penis." This may consist of an emission of semen, leakage or slight moisture at the opening of the meatus or of seminal products. These discharges may be due to gonorrhea or to poison developed in the vagina of rank women or a concealed chancre in the urethra, or they may be non-specific as from stricture, urethritis, vesiculitis, low fevers or prostatic catarrh. Specific or venereal disease is not a part of this paper and is used in the sense of cause and effect. Seminal weakness and true spermatorrhea frequently depend on abnormal conditions that belong to surgery. This class of patients so often come under the care of the general practitioner that all should be familiar with the frequency and importance of this disease.

Involuntary seminal emissions occurring during sleep in persons from 17 to 40 years of age is not of rare occurrence. These frequently take place during an erotic dream. An emission may occur without voluntary effort either normal or abnormal. When the emissions are at entervals of a few weeks or more they are not pathological; although this class of persons are often needlessly alarmed. Especially is this the case if they have been reading books and lectures on this subject, that are scattered broadcast to reap revenue. Hence the mind of such patients is much disturbed and if positive assurance on the part of the physician is withheld, the general health of such patients may be undermined as though they were suffering from the real disease. The medical adviser is often put to the test whether he will honor his profession or degrade it. When seminal emissions take place more frequently than stated above they are to be regarded as pathological, which may be associated with true spermatorrhea; the semen passing into the bladder is voided with the urine or is discharged when the feces are passed. The urine in true spermatorrhea always contains spermatozoa. It is asserted that a urethral discharge from an adult male of ten days duration will contain spermatozoa. The microscope will determine this vexed question. It is safe to assert that all urethral leaks should be promptly arrested. Back of this will be found a devitalized condition of the system as a result of masturbation, coitus with lewd women of the brothel type, sexual excess, neurasthenia, bicycle exercise, railroad life, especially engine drivers and conductors, also disease of the veins of the testicles which impairs the nutrition of these organs, leading to atrophy and partial or complete impotence. There are far too many men that are sexually weak; especially is this the case in our large cities. Such men cannot procreate a healthy progeny, and it is telling on the blood of the nation. Can we imagine Rachels weeping for children that their sterile husbands can not procreate? An authority says it takes four or five days for the spermatozoa in a man to mature. When this vital fluid is continually drained off, a father, if fertile at all, will have an offspring that will be weakly and probably of short life. These are questions that need the proper solution for the good of the race. Truly "the sins of the fathers are visited upon their children."

The intelligent stock-breeder puts the ban on a masturbating or sexually weak sire. Is the father of a family not of more importance than a bull or a stallion? To prove that sexual weakness is transmitted I will mention a case in point. A law student applied to me last winter for treatment. He was suffering with seminal weakness in a marked degree; aged 24 years, medium size, well nourished. Mental condition only fair. Had suffered from seminal emissions for six or seven years. The last two or three years the emissions had become frequent, they were diurnal and nocturnal. Said when in the company of ladies he was very much annoyed with involuntary discharges of semen. He had masturbated for years and had an exceedingly tender urethra. This young man's father and oldest brother are reputable physicians and they are sexually weak. Many of the physical and mental wrecks of young men and women are due to sexual excess.

*Treatment.*—This will consist of proper medication, surgery, hygiene, moral and honest cooperation of the patient. The bromides, iron, phosphorus, bitter tonics and electricity have been highly commended. Of recent years I have been prescribing the f. e. of black willow, a teaspoonful three or four times a day. The cure will be hastened by the additional use of suppositories and bougies of the black willow to be inserted at bedtime each alternate night. The black willow is our best remedy to seal up seminal leaks; being a sedative tonic and astringent to the seminal vesicles, ejacular ducts, veins and arteries. Select a good article and push it with confidence. If the patient is very nervous and sleeping badly, give the following recipe: f. e. black willow, tr. green root gelsem., f. e. passiflora incarnata, equal parts; give one-half to a teaspoonful four times a day. I regard this as a happy blending of drugs, nothing better to keep the penis under subordination. Regulate the bowels and improve the general health. The brain and nerve cells, like impoverished soil, need good fertilizers, like protonuclein, kephalin and concentrated tr. of oats. In the more serious cases give thyroid extract and spermin.

The surgery will mostly consist of passing bougies of increasing size once or twice a week. The urethra will nearly always show unusual tenderness and hyperesthesia. Hence the greatest gentleness should be used in all manipulations with the urethra in this class of cases, or they will rebel against this essential part of the treatment. In some cases it will be best to cocain the urethra. The value of injections into the prostatic portion of the urethra for the cure of spermatorrhea was pointed out long ago by Lalleman; he used strong solutions of nitrate of silver. I think milder injections the better treatment. Circumcision may be necessary in some cases. The hygiene will consist of a firm bed, light covering; sleep on the right side, bathe spine and genital organs with cool water twice a day. A plain but nutritious diet should be enjoined with outdoor exercise. Alcohol and tobacco should be abstained from.

The moral and cooperative treatment must be insisted upon, for without it the physician cannot prove his skill and the patient cannot be cured. The patient must be filled with hope, higher aspirations and a better manhood. Tell him you can cure him if he will stop his sexual vices and faithfully carry out your instructions. Impress upon the patient the importance of chaste associates, clean literature and pictures. The question will be asked, should this class of persons marry? Yes, when cured and fit to be husbands. I cannot close this important subject without a solemn plea for greater prophylactic care of children. Proper instructions to young men and women that they may escape some of the pitfalls of early sexual abuse.

### THE KNOT IN THE STRING OF THE APRON OF MOTHER EDDY.

Mother Mary Baker Glover Patterson Eddy has lately forbidden her children to "doctor" infectious and contagious diseases. Let us invoke upon her irresponsible brood the grace of obedience.

This prohibition follows upon the heels of the indictment of a famous "healer" in New York for the manslaughter of a believer's child. The inconsiderate action of the magistrate in this case indicated to Mother Eddy a sad lack of information on the rights and powers of Christian Science, and justified, in her opinion, a restriction of the zeal of her disciples. Therefore the Prophetess shortened her apron-strings, and "until human thought is better informed," Christian Scientists must refrain from "doctoring" infectious diseases. The pontifical apron-strings were once before adjusted to human misinformation. About two years ago Mother Eddy made an edict in which she said that Christian Science is the very best surgeon, but that the demonstration of this preeminence would be reserved for a later generation. The faithful were therefore advised to address themselves exclusively to the minds of their injured patients, leaving the injuries to the fingers of the surgeons. In its frank attribution of fingers to the surgeon Mrs. Eddy's edict was an example of that delicious sort of humor which is never conceived in the mind of a humorist.

It is somewhat doubtful if the dear children will heed their wise mother's later injunction. They did not, at any rate, refrain from "doctoring" surgical cases.

An aged lady who lived near the writer fell and injured her hip quite severely. Her two daughters, both past middle life, were "healers," and undertook to cure their mother. The neighbors often saw on the streets the two healers conducting a very aged woman whose efforts to walk were accompanied with every evidence of exquisite torture. The deluded daughters were totally blind to their mother's agony. They took her to a watering-place where they continued to dispute the "claim" in the same excruciating manner. But intracapsular fracture is a stubborn "claim," and at length the poor sufferer went to rest. A few months later the elder sister yielded to a "claim," and followed her mother. The one survivor moved from the locality.

If the triumph of faith over reason has been indefinitely postponed with respect to surgical cases and infectious diseases, the demonstrations of Christian Science can hardly be spectacular, and, what is worse, they must depreciate in cash value. But the most discouraging feature of the situation is the confessed abeyance of Christian Science to "human thought." When "human thought is better informed" infectious diseases and surgical cases may both be treated by Christian Science. How human thought is to become better informed does not appear, but certainly not by demonstrations, for the demonstrations which have been and those which might have been are all abandoned. Mortal mind seems to be perversely and persistently acquiring misinformation, and since Mother Eddy's apron-strings have already been twice shortened out of respect to human misinformation, it is conceivable at least that the liberty of the faithful may again be curtailed.

In abandoning the past performances of Christian Science in the treatment of infectious diseases complete sacrifice is made of all the pitiful offerings which have been in the belief that the Word of healing has indeed been vouchsafed to men. What a shock to those who hold this fond delusion to be told that in the face of an unbelieving majority the Good Mother will withhold the Gift even from believers!—*Maryland Medical Journal.*

---

### EDUCATIONAL LIMITATION OF VENEREAL DISEASES.*

F. C. Valentine, of New York, recapitulates his ideas on this question as follows: (1) Sufficient of the physiology and pathology of the genito-urinary apparatus should be taught in institutions for higher education to convey to students the dangers of genito-urinary diseases to themselves and to others. (2) Similar instructions should be given in schools attended by boys at the age of puberty. (3) No man who has ever had gonorrhea should be allowed to marry until it is proved by a physician that he can not infect his wife. (4) Regular physicians should be elected by their societies to deliver evening lectures to the public on genito-urinary diseases. (5) Every father should be taught to warn his sons of

N. Y. Med. Rec., Nov. 8, 1902.

the dangers of genito-urinary diseases. When, from incompetency or delicacy, the father can not or does not wish to do this, the family physician should discharge that duty. (6) Every medical society should elect its most competent member to write at least one article on the subject, worded for laymen's comprehension, and to be published under the auspices of the society. The segregation of prostitutes would free reputable neighborhoods from the evil influences, bad example, demoralization, and infection of this social disease. It would also be of assistance in the enforcement of the proposed laws, and would likewise tend to suppress assignation houses, which are nothing but conveniences for clandestine prostitutes, the worst disseminators of venereal diseases. The concentration of prostitutes in districts set aside for them would facilitate examination as to their ability to infect others. Such examinations should be made every day, as an approach to effective work. Under the present state of affairs, the inefficiency of registration and periodical examination is due principally to these two facts, viz.: A prostitute can become infected and transmit the infection to many men during the intervals between examinations. The clandestine prostitute can disseminate venereal disease wholly without restraint under the best system of registration and examination, which she escapes.—*St. Louis Medical Review.*

---

### THE USE OF PARAFFIN FOR RESTORING THE BRIDGE OF THE NOSE.

Stephen Paget reports two successful cases. In the first case, a paraffin (Eckstein's) was used, that had a melting point of 136° F. In the second, a paraffin with a melting point of 115°. The following considerations are to be noted:

1. Whatever may be the melting point of the paraffin, it must be kept, during use, 10° or 15° higher, or it will solidify in the needle before it can be injected.

2. For the same reason, the needle and syringe must be kept 15° or 20° higher than the paraffin. The loss of heat from the syringe may be to some extent checked by casing it in a bit of drainage-tube.

3. An ordinary glass antitoxin syringe, with a well-fitting asbestos piston, answers every purpose. The needle must be broad and strong, such as is used for exploring the pleural cavity; but the needle generally used for this purpose is too long, and should be shortened to an inch and a half.

4. An assistant must make firm pressure, very carefully, all round the nose, and must keep up this pressure till the paraffin is set. But it sets almost at once, allowing only a quarter or half a minute to the surgeon to mould it. The firm pressure may be helped by the use of a strip of lead or pewter under the tips of the fingers.

5. The skin of the nose, at the point where the needle is to enter, should be nicked with a scalpel, so that the needle may pass easily. It is best to direct the needle downward, away from the eyelids, and to introduce it at the middle line of the nose.

6. Eckstein's paraffin, melting at 136°, is difficult to use, and must be very quickly transferred from the bottle into the subcutaneous tissue. Probably it is best suited for a case where only a very small quantity of paraffin is required. With this paraffin the syringe must be kept so hot that it can hardly be handled except with gloves.

7. One case has been recorded of sloughing of the skin, presumably from heat. This disaster could hardly happen with paraffin melting at 115°. More than one case has been recorded where signs attributable to pulmonary embolism followed the injection. It is, therefore, necessary to avoid piercing a vein, and to keep firm and close pressure all round the nose during and after the injection.

8. After the treatment a fold of lint should be kept over the upper part of the face, and kept moist with cold or iced lotion. In the two cases quoted there was little or no pain after the operation, but some swelling round the nose for three or four days.

It is, of course, impossible by any method to make a narrow or refined bridge to the nose in these cases. The front view of it is still heavy and clumsy, but the profile view is excellent, and this good result is obtained without any scar and without any severe or difficult operation, in a few minutes.—*St. Louis Medical Review.*

